# Crystal structure of bis­{4-[(4-methyl­benz­yl)­oxy]-*N*′-(4-methyl­benzyl­idene)benzohydrazidato}nickel(II)

**DOI:** 10.1107/S2056989022009392

**Published:** 2022-09-30

**Authors:** Md. Hasan Al Banna, Md. Belayet Hossain Howlader, Ryuta Miyatake, Md. Chanmiya Sheikh, Ennio Zangrando

**Affiliations:** a Department of Chemistry, Rajshahi University, Rajshahi-6205, Bangladesh; b Center for Environmental Conservation and Research Safety, University of Toyama, 3190 Gofuku, Toyama, 930-8555, Japan; c Department of Applied Science, Faculty of Science, Okayama University of Science, Japan; d Department of Chemical and Pharmaceutical SCiences, University of Trieste, Italy; Vienna University of Technology, Austria

**Keywords:** crystal structure, nickel(II) complex, square-planar coordination, aroylhydrazone ligand

## Abstract

In the title complex, [Ni(C_23_H_21_N_2_O_2_)_2_], the central Ni^II^ atom is located on an inversion centre and exhibits a slightly distorted square-planar N_2_O_2_ coordination environment. A *trans-*configuration of the *N*,*O* chelating ligands results from the imposed site symmetry of the central Ni^II^ atom. In the crystal, individual mol­ecules stack along the *a* axis through weak π–π stacking inter­actions between the phenyl rings.

## Abstract 2

In the title compound, the mononuclear nickel(II) complex exhibits point group symmetry 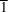 .

## 1. Chemical context

Variously substituted hydrazone ligands have attracted special attention because of their chelating capabilities and structural properties, such as the degree of rigidity, a conjugated π-system and an N—H unit that readily participates in hydrogen bonding and may be easily deprotonated. The corresponding nickel(II) complexes are of considerable inter­est since they exhibit a broad spectrum of physiological and pharmacological activities (Yang *et al.*, 2020 ▸; Al-Qadsy *et al.*, 2021 ▸; Neethu *et al.*, 2021 ▸; Krishnamoorthy *et al.*, 2012 ▸), most of which are structure-dependent properties. 
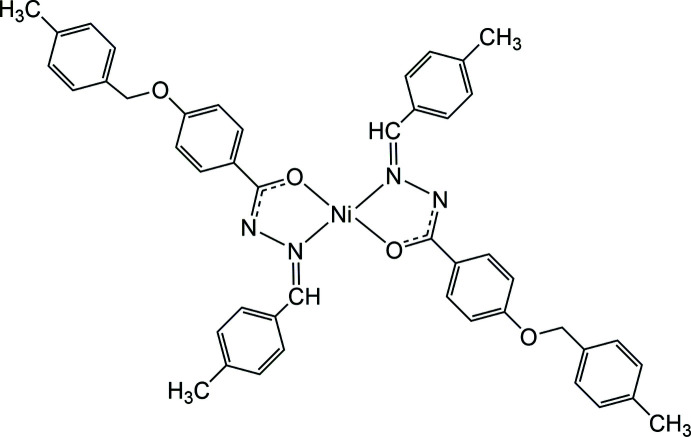


We report here the synthesis and crystal structure of another Ni^II^ complex with a derivatized hydrazone ligand.

## 2. Structural commentary

**Figure 1:**
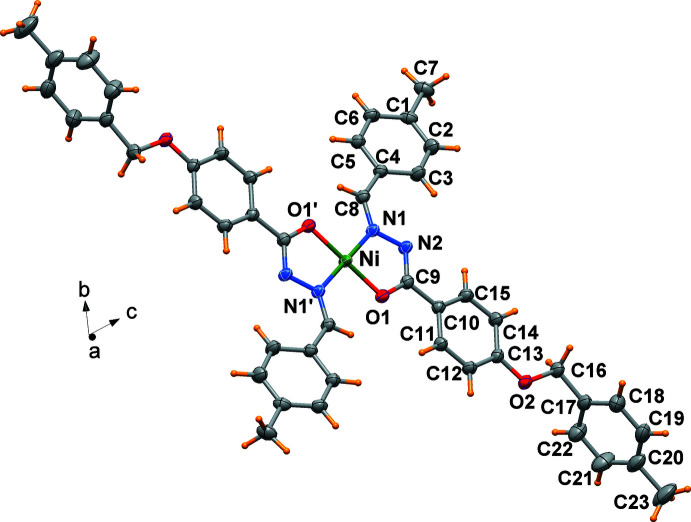
Mol­ecular structure of the centrosymmetric nickel(II) complex, drawn with displacement ellipsoids at the 50% probability level. [symmetry code for primed atoms: −*x* + 2, −*y*, −*z* + 2.]

The central metal Ni^II^ atom of the title complex is located on an inversion center. Hence, the asymmetric unit comprises half a mol­ecule (Fig. 1 ▸). The enolizable O atom and the azometh­ine N atom of the ligand coordinate to the Ni^II^ atom to form a five-membered chelate ring. The Ni^II^ atom exhibits a slightly distorted square-planar coordination environment with the deprotonated ligands in a *trans* configuration imposed by the crystal symmetry. The Ni—N1 and Ni—O1 bond lengths are 1.8677 (12) and 1.8363 (10) Å, respectively, with a chelating angle of 83.47 (5)°. These data are in agreement with previously reported crystal structures of related complexes (Yang *et al.*, 2020 ▸; Al-Qadsy *et al.*, 2021 ▸; Neethu *et al.*, 2021 ▸; Krishnamoorthy *et al.*, 2012 ▸), irrespective of the substituents present in the ligand.

As expected, the C9—O1 bond length of 1.3009 (18) Å lies between a C—O single bond (1.43 Å; Allen *et al.*, 1987 ▸) and a C=O double bond (1.21 Å; Allen *et al.*, 1987 ▸). The bond lengths N1—C8 of 1.2977 (19) Å and N2—C9 of 1.3145 (18) Å are close to the value of a typical C=N bond (1.30 Å; Allen *et al.*, 1987 ▸). These data reveal that the —CH=N—N=C—O fragment of the ligand remains a conjugated system even after the loss of a H atom from its enolized carbonyl O atom. The complex is stabilized by weak intra­molecular C8—H8⋯O1, C3—H3⋯N2 and C11—H11⋯O1 hydrogen bonds involving phenyl and methylene donor groups and the coordinating atoms as acceptor groups (Table 1 ▸). The benzyl­idene ring is tilted by 26.06 (6)° with respect to the N_2_O_2_ coordination plane, while the phenyl rings of the ether moiety form a dihedral angle of 83.29 (5)°.

The bond-valence sum (BVS) calculated for the Ni^II^ atom present in the complex, using the parameters of Brese & O’Keeffe (1991 ▸), indicate a higher value (2.97 valence units) than expected for a formal ionic charge of +2. The calculated high value can be reasonably attributed to a very pronounced covalent bonding associated with the Ni—O and Ni—N bonds. As a matter of fact, a set of new optimized *r*_0_ parameters to be used for the BVS calculation for model compounds involving Ni^II^—O, Ni^II^—S, Ni^II^—N inter­actions has been proposed (Liu & Thorp, 1993 ▸). By using these values, the BVS calculation for this complex gives a value of 2.36 valence units.

## 3. Supra­molecular features

**Figure 2:**
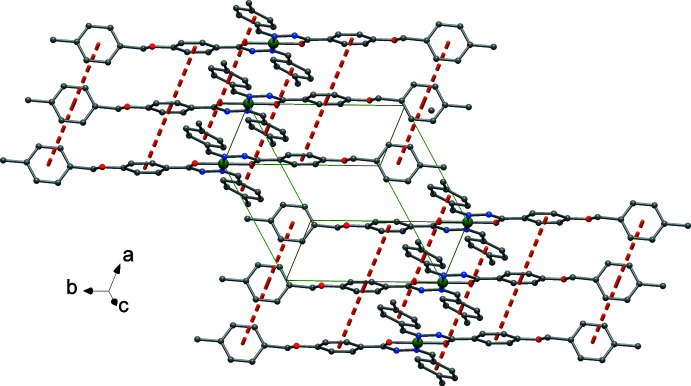
Crystal packing of individual complexes showing the π-ring inter­actions as dotted lines.

Individual mol­ecular complexes are weakly packed along the *a* axis through π-ring inter­actions involving the phenyl rings, with centroid-to-centroid distances of 4.6914 (2) Å and a slippage of *ca* 3.0-3.3 Å, as shown in Fig. 2 ▸. In addition, the five-membered chelate rings of neighbouring complexes have even shorter distances [3.4555 (2) Å with a slippage of 0.96 Å].

## 4. Database survey

A search in the Cambridge Crystal Structure Database (CSD, version 5.43, update June 2022); Groom *et al.*, 2016 ▸) retrieved more than twenty bis-chelated square-planar nickel(II) complexes with hydrazone-based ligands also bearing bulky ferrocenyl groups (Krishnamoorthy *et al.*, 2012 ▸), 2,2′-bi­thio­phenyl (Yang *et al.*, 2020 ▸) or 9-anthryl­methyl­ene fragments (Mondal *et al.*, 2014 ▸). However, no species comprising a long benzyl-phenyl ether chain has been reported so far. It is worth noting that all characterized Ni^II^ complexes exhibit a *trans*-configuration of ligands, where the —CH=N—N=C—O fragment is chelating, and the coordination Ni—O and Ni—N bond lengths do not appear to be significantly affected by the electronic or steric properties of groups present on the ligands.

## 5. Synthesis and crystallization

To a solution of 4-(4-methyl­benz­yloxy)benzoyl­hydrazine (0.26 g, 1 mmol in 25 ml of ethanol), 4-methyl benzaldehyde (0.12 g, 1 mmol) was added and the mixture was refluxed for half an hour. A solution of nickel(II) acetate tetra­hydrate (0.13 g, 0.5 mmol in 5 ml of ethanol) was then added and refluxing was continued for 2 h. The obtained orange precipitate was filtered off and washed three times with hot ethanol. The product was recrystallized from a mixture of chloro­form and aceto­nitrile (5:1, *v*/*v*) and orange crystals, suitable for X-ray diffraction, were filtered off, washed with hot ethanol, and left to dry in a desiccator over silica gel. Yield: 0.45 g, 58%. Melting point: >523 K. FT–IR: 1603, 1585 ν (C=N—N=C), 486 ν (*M*—N), 503 ν (*M*—O). LC–MS (ESI) *m*/*z*: [*M* + H]^+^. Calculated for C_46_H_42_N_4_O_4_Ni 773.2632; found 773.2636. μ_eff_: 0.832 B·M. Molar conductance (ohm^−1^ cm^2^ mol^−1^): 1.0. NMR spectra were not obtained due to the low solubility of the complex even in DMSO.

## 6. Refinement

Crystal data, data collection and structure refinement details are summarized in Table 2 ▸. The hydrogen atoms were included in idealized positions as riding contributions with fixed isotropic displacement parameters [C—H = 0.95–0.99 Å; *U*_iso_(H) = 1.2 or 1.5 *U*_eq_(C)].

## Supplementary Material

Crystal structure: contains datablock(s) global, I. DOI: 10.1107/S2056989022009392/wm5661sup1.cif

Structure factors: contains datablock(s) I. DOI: 10.1107/S2056989022009392/wm5661Isup2.hkl

CCDC reference: 2174697

Additional supporting information:  crystallographic information; 3D view; checkCIF report

## Figures and Tables

**Table 1:** Hydrogen-bond geometry (Å, °)

*D*—H⋯*A*	*D*—H	H⋯*A*	*D*⋯*A*	*D*—H⋯*A*
C8—H8⋯O1^i^	0.95	2.38	2.9455 (18)	118
C3—H3⋯N2	0.95	2.37	2.945 (2)	118
C11—H11⋯O1	0.95	2.43	2.7590 (19)	100

Symmetry code: (i) 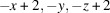 .

**Table 2:** Experimental details

Crystal data	
Chemical formula	[Ni(C_23_H_21_N_2_O_2_)_2_]
*M*_r_	773.54
Crystal system, space group	Triclinic, *P* 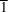
Temperature (K)	173
*a*, *b*, *c* (Å)	4.6914 (2), 13.0677 (7), 16.9923 (8)
α, β, γ (°)	68.441 (5), 83.739 (6), 88.032 (6)
*V* (Å^3^)	963.05 (9)
*Z*	1
Radiation type	Mo *K*α
μ (mm^−1^)	0.55
Crystal size (mm)	0.32 × 0.08 × 0.03

Data collection	
Diffractometer	Rigaku R-AXIS RAPID
Absorption correction	Multi-scan (*ABSCOR*; Rigaku, 1995 ▸)
*T*_min_, *T*_max_	0.761, 0.984
No. of measured, independent and observed [*I* > 2σ(*I*)] reflections	9456, 4375, 3883
*R*_int_	0.024
(sin θ/λ)_max_ (Å^−1^)	0.649

Refinement	
*R*[*F* ^2^ > 2σ(*F* ^2^)], *wR*(*F* ^2^), *S*	0.037, 0.096, 1.06
No. of reflections	4375
No. of parameters	252
H-atom treatment	H-atom parameters constrained
Δρ_max_, Δρ_min_ (e Å^−3^)	0.42, −0.19

Computer programs: *CrystalStructure* (Rigaku, 2018 ▸), *SHELXT* (Sheldrick, 2015*a* ▸), *SHELXL* (Sheldrick, 2015*b* ▸), *DIAMOND* (Brandenburg, 1999 ▸) and *WinGX* (Farrugia, 2012 ▸).

## supplementary crystallographic information

### Crystal data

**Table:**

[Ni(C_23_H_21_N_2_O_2_)_2_]	*Z* = 1
*M**_r_* = 773.54	*F*(000) = 406
Triclinic, *P*1	*D*_x_ = 1.334 Mg m^−^^3^
*a* = 4.6914 (2) Å	Mo *K*α radiation, λ = 0.71075 Å
*b* = 13.0677 (7) Å	Cell parameters from 8457 reflections
*c* = 16.9923 (8) Å	θ = 1.7–27.5°
α = 68.441 (5)°	µ = 0.55 mm^−^^1^
β = 83.739 (6)°	*T* = 173 K
γ = 88.032 (6)°	Platelet, orange
*V* = 963.05 (9) Å^3^	0.32 × 0.08 × 0.03 mm

### Data collection

**Table:**

Rigaku R-AXIS RAPID diffractometer	3883 reflections with *I* > 2σ(*I*)
Detector resolution: 10.000 pixels mm^-1^	*R*_int_ = 0.024
ω scans	θ_max_ = 27.5°, θ_min_ = 2.5°
Absorption correction: multi-scan (ABSCOR; Rigaku, 1995)	*h* = −6→5
*T*_min_ = 0.761, *T*_max_ = 0.984	*k* = −16→16
9456 measured reflections	*l* = −22→22
4375 independent reflections	

### Refinement

**Table:**

Refinement on *F*^2^	0 restraints
Least-squares matrix: full	Hydrogen site location: inferred from neighbouring sites
*R*[*F*^2^ > 2σ(*F*^2^)] = 0.037	H-atom parameters constrained
*wR*(*F*^2^) = 0.096	*w* = 1/[σ^2^(*F*_o_^2^) + (0.0564*P*)^2^ + 0.1727*P*] where *P* = (*F*_o_^2^ + 2*F*_c_^2^)/3
*S* = 1.06	(Δ/σ)_max_ = 0.006
4375 reflections	Δρ_max_ = 0.42 e Å^−^^3^
252 parameters	Δρ_min_ = −0.19 e Å^−^^3^

### Special details

**Table:**

Geometry. All esds (except the esd in the dihedral angle between two l.s. planes) are estimated using the full covariance matrix. The cell esds are taken into account individually in the estimation of esds in distances, angles and torsion angles; correlations between esds in cell parameters are only used when they are defined by crystal symmetry. An approximate (isotropic) treatment of cell esds is used for estimating esds involving l.s. planes.

### Fractional atomic coordinates and isotropic or equivalent isotropic displacement parameters (Å^2^)

**Table:**

	*x*	*y*	*z*	*U*_iso_*/*U*_eq_	
Ni1	1.000000	0.000000	1.000000	0.02390 (10)	
O1	0.8708 (2)	0.13421 (8)	0.93185 (7)	0.0267 (2)	
O2	0.2680 (3)	0.52033 (9)	0.65888 (7)	0.0339 (3)	
N1	0.7701 (3)	−0.05385 (10)	0.94061 (8)	0.0250 (3)	
N2	0.6329 (3)	0.02767 (10)	0.87729 (8)	0.0272 (3)	
C1	0.1840 (3)	−0.32649 (13)	0.85447 (11)	0.0313 (3)	
C2	0.2533 (4)	−0.21651 (14)	0.80907 (11)	0.0389 (4)	
H2	0.183365	−0.181019	0.755246	0.047*	
C3	0.4201 (4)	−0.15681 (13)	0.83933 (11)	0.0354 (4)	
H3	0.457644	−0.081018	0.807386	0.042*	
C4	0.5340 (3)	−0.20830 (12)	0.91727 (10)	0.0267 (3)	
C5	0.4694 (4)	−0.31917 (12)	0.96220 (10)	0.0302 (3)	
H5	0.546693	−0.355980	1.014716	0.036*	
C6	0.2951 (4)	−0.37703 (13)	0.93214 (11)	0.0325 (3)	
H6	0.250866	−0.452077	0.964938	0.039*	
C7	−0.0032 (4)	−0.38838 (15)	0.82047 (13)	0.0408 (4)	
H7A	−0.191485	−0.353241	0.813325	0.049*	
H7B	−0.026408	−0.464591	0.860552	0.049*	
H7C	0.086306	−0.387620	0.765383	0.049*	
C8	0.7190 (3)	−0.15674 (12)	0.95597 (10)	0.0269 (3)	
H8	0.818971	−0.207025	0.999831	0.032*	
C9	0.7016 (3)	0.12306 (12)	0.87971 (9)	0.0248 (3)	
C10	0.5783 (3)	0.22452 (12)	0.82102 (9)	0.0246 (3)	
C11	0.6537 (4)	0.32665 (12)	0.82264 (10)	0.0285 (3)	
H11	0.782209	0.329648	0.861251	0.034*	
C12	0.5427 (4)	0.42268 (12)	0.76866 (10)	0.0311 (3)	
H12	0.592488	0.491322	0.770952	0.037*	
C13	0.3584 (3)	0.41942 (12)	0.71090 (9)	0.0272 (3)	
C14	0.2802 (4)	0.31891 (13)	0.70838 (10)	0.0300 (3)	
H14	0.154229	0.316308	0.669017	0.036*	
C15	0.3889 (4)	0.22253 (12)	0.76416 (10)	0.0289 (3)	
H15	0.332585	0.153781	0.763416	0.035*	
C16	0.1042 (4)	0.52370 (13)	0.59118 (10)	0.0338 (4)	
H16A	−0.077691	0.482264	0.614859	0.041*	
H16B	0.214098	0.489949	0.553892	0.041*	
C17	0.0433 (4)	0.64249 (13)	0.54117 (10)	0.0342 (4)	
C18	0.1936 (5)	0.69725 (15)	0.46333 (12)	0.0467 (5)	
H18	0.341540	0.660436	0.441520	0.056*	
C19	0.1304 (6)	0.80648 (16)	0.41616 (13)	0.0533 (5)	
H19	0.235121	0.842724	0.362308	0.064*	
C20	−0.0782 (5)	0.86203 (16)	0.44579 (14)	0.0549 (6)	
C21	−0.2279 (6)	0.80699 (18)	0.52500 (17)	0.0641 (6)	
H21	−0.372618	0.844499	0.547317	0.077*	
C22	−0.1685 (5)	0.69813 (16)	0.57170 (14)	0.0490 (5)	
H22	−0.274742	0.661553	0.625238	0.059*	
C23	−0.1500 (7)	0.98005 (19)	0.3935 (2)	0.0871 (10)	
H23A	−0.100061	1.028406	0.422521	0.104*	
H23B	−0.355632	0.985730	0.387091	0.104*	
H23C	−0.040649	1.002234	0.337299	0.104*	

### Atomic displacement parameters (Å^2^)

**Table:**

	*U*^11^	*U*^22^	*U*^33^	*U*^12^	*U*^13^	*U*^23^
Ni1	0.02565 (16)	0.01922 (14)	0.02805 (16)	0.00079 (10)	−0.01098 (11)	−0.00788 (11)
O1	0.0289 (6)	0.0218 (5)	0.0305 (5)	0.0003 (4)	−0.0119 (4)	−0.0083 (4)
O2	0.0470 (7)	0.0234 (5)	0.0318 (6)	0.0040 (5)	−0.0195 (5)	−0.0069 (5)
N1	0.0265 (6)	0.0217 (6)	0.0269 (6)	0.0018 (5)	−0.0089 (5)	−0.0073 (5)
N2	0.0305 (7)	0.0212 (6)	0.0303 (6)	0.0023 (5)	−0.0131 (5)	−0.0074 (5)
C1	0.0277 (8)	0.0324 (8)	0.0412 (9)	0.0015 (6)	−0.0071 (7)	−0.0214 (7)
C2	0.0479 (11)	0.0329 (8)	0.0393 (9)	0.0032 (8)	−0.0218 (8)	−0.0128 (7)
C3	0.0457 (10)	0.0248 (7)	0.0360 (9)	−0.0020 (7)	−0.0170 (7)	−0.0077 (7)
C4	0.0287 (8)	0.0234 (7)	0.0306 (8)	0.0018 (6)	−0.0084 (6)	−0.0117 (6)
C5	0.0353 (9)	0.0253 (7)	0.0313 (8)	0.0008 (6)	−0.0093 (7)	−0.0106 (6)
C6	0.0363 (9)	0.0252 (7)	0.0376 (8)	−0.0041 (6)	−0.0050 (7)	−0.0126 (7)
C7	0.0373 (10)	0.0442 (10)	0.0525 (11)	−0.0026 (8)	−0.0119 (8)	−0.0291 (9)
C8	0.0282 (8)	0.0235 (7)	0.0297 (7)	0.0019 (6)	−0.0097 (6)	−0.0088 (6)
C9	0.0236 (7)	0.0245 (7)	0.0264 (7)	0.0004 (6)	−0.0054 (6)	−0.0086 (6)
C10	0.0251 (7)	0.0229 (7)	0.0252 (7)	0.0010 (6)	−0.0052 (6)	−0.0075 (6)
C11	0.0324 (8)	0.0256 (7)	0.0294 (8)	0.0010 (6)	−0.0114 (6)	−0.0100 (6)
C12	0.0396 (9)	0.0226 (7)	0.0328 (8)	−0.0003 (6)	−0.0126 (7)	−0.0098 (6)
C13	0.0308 (8)	0.0235 (7)	0.0251 (7)	0.0025 (6)	−0.0069 (6)	−0.0055 (6)
C14	0.0333 (8)	0.0275 (7)	0.0316 (8)	0.0016 (6)	−0.0142 (7)	−0.0107 (6)
C15	0.0318 (8)	0.0236 (7)	0.0329 (8)	−0.0010 (6)	−0.0095 (6)	−0.0103 (6)
C16	0.0415 (10)	0.0290 (8)	0.0323 (8)	0.0039 (7)	−0.0175 (7)	−0.0095 (7)
C17	0.0419 (10)	0.0281 (8)	0.0329 (8)	0.0019 (7)	−0.0176 (7)	−0.0079 (7)
C18	0.0639 (13)	0.0373 (10)	0.0352 (9)	0.0030 (9)	−0.0061 (9)	−0.0090 (8)
C19	0.0788 (16)	0.0381 (10)	0.0358 (10)	−0.0077 (10)	−0.0143 (10)	−0.0021 (8)
C20	0.0710 (15)	0.0297 (9)	0.0589 (13)	0.0028 (9)	−0.0322 (11)	−0.0033 (9)
C21	0.0626 (15)	0.0408 (11)	0.0793 (16)	0.0178 (10)	−0.0088 (13)	−0.0120 (11)
C22	0.0490 (12)	0.0379 (10)	0.0505 (11)	0.0070 (9)	−0.0047 (9)	−0.0055 (9)
C23	0.111 (2)	0.0359 (12)	0.097 (2)	0.0088 (13)	−0.0446 (19)	0.0047 (13)

### Geometric parameters (Å, º)

**Table:**

Ni1—O1^i^	1.8363 (10)	C10—C15	1.389 (2)
Ni1—O1	1.8363 (10)	C10—C11	1.403 (2)
Ni1—N1	1.8677 (12)	C11—C12	1.378 (2)
Ni1—N1^i^	1.8678 (12)	C11—H11	0.9500
O1—C9	1.3009 (18)	C12—C13	1.390 (2)
O2—C13	1.3736 (17)	C12—H12	0.9500
O2—C16	1.4383 (18)	C13—C14	1.392 (2)
N1—C8	1.2977 (19)	C14—C15	1.389 (2)
N1—N2	1.4030 (16)	C14—H14	0.9500
N2—C9	1.3145 (18)	C15—H15	0.9500
C1—C2	1.389 (2)	C16—C17	1.506 (2)
C1—C6	1.391 (2)	C16—H16A	0.9900
C1—C7	1.504 (2)	C16—H16B	0.9900
C2—C3	1.382 (2)	C17—C18	1.376 (3)
C2—H2	0.9500	C17—C22	1.378 (3)
C3—C4	1.402 (2)	C18—C19	1.396 (3)
C3—H3	0.9500	C18—H18	0.9500
C4—C5	1.393 (2)	C19—C20	1.360 (3)
C4—C8	1.460 (2)	C19—H19	0.9500
C5—C6	1.385 (2)	C20—C21	1.393 (3)
C5—H5	0.9500	C20—C23	1.518 (3)
C6—H6	0.9500	C21—C22	1.386 (3)
C7—H7A	0.9800	C21—H21	0.9500
C7—H7B	0.9800	C22—H22	0.9500
C7—H7C	0.9800	C23—H23A	0.9800
C8—H8	0.9500	C23—H23B	0.9800
C9—C10	1.479 (2)	C23—H23C	0.9800
			
O1^i^—Ni1—O1	180.0	C12—C11—C10	120.54 (14)
O1^i^—Ni1—N1	96.53 (5)	C12—C11—H11	119.7
O1—Ni1—N1	83.47 (5)	C10—C11—H11	119.7
O1^i^—Ni1—N1^i^	83.47 (5)	C11—C12—C13	120.22 (14)
O1—Ni1—N1^i^	96.53 (5)	C11—C12—H12	119.9
N1—Ni1—N1^i^	180.00 (5)	C13—C12—H12	119.9
C9—O1—Ni1	111.02 (9)	O2—C13—C12	114.99 (13)
C13—O2—C16	117.77 (12)	O2—C13—C14	124.86 (14)
C8—N1—N2	119.42 (12)	C12—C13—C14	120.15 (14)
C8—N1—Ni1	125.97 (11)	C15—C14—C13	119.15 (14)
N2—N1—Ni1	114.52 (9)	C15—C14—H14	120.4
C9—N2—N1	107.12 (12)	C13—C14—H14	120.4
C2—C1—C6	117.53 (15)	C14—C15—C10	121.40 (14)
C2—C1—C7	121.02 (16)	C14—C15—H15	119.3
C6—C1—C7	121.45 (15)	C10—C15—H15	119.3
C3—C2—C1	122.41 (16)	O2—C16—C17	107.78 (13)
C3—C2—H2	118.8	O2—C16—H16A	110.2
C1—C2—H2	118.8	C17—C16—H16A	110.2
C2—C3—C4	119.88 (15)	O2—C16—H16B	110.2
C2—C3—H3	120.1	C17—C16—H16B	110.2
C4—C3—H3	120.1	H16A—C16—H16B	108.5
C5—C4—C3	117.81 (14)	C18—C17—C22	118.55 (17)
C5—C4—C8	116.21 (13)	C18—C17—C16	120.82 (17)
C3—C4—C8	125.97 (14)	C22—C17—C16	120.61 (16)
C6—C5—C4	121.58 (15)	C17—C18—C19	120.5 (2)
C6—C5—H5	119.2	C17—C18—H18	119.7
C4—C5—H5	119.2	C19—C18—H18	119.7
C5—C6—C1	120.75 (15)	C20—C19—C18	121.3 (2)
C5—C6—H6	119.6	C20—C19—H19	119.3
C1—C6—H6	119.6	C18—C19—H19	119.3
C1—C7—H7A	109.5	C19—C20—C21	118.14 (18)
C1—C7—H7B	109.5	C19—C20—C23	121.1 (2)
H7A—C7—H7B	109.5	C21—C20—C23	120.7 (2)
C1—C7—H7C	109.5	C22—C21—C20	120.8 (2)
H7A—C7—H7C	109.5	C22—C21—H21	119.6
H7B—C7—H7C	109.5	C20—C21—H21	119.6
N1—C8—C4	130.93 (14)	C17—C22—C21	120.6 (2)
N1—C8—H8	114.5	C17—C22—H22	119.7
C4—C8—H8	114.5	C21—C22—H22	119.7
O1—C9—N2	123.84 (13)	C20—C23—H23A	109.5
O1—C9—C10	117.23 (12)	C20—C23—H23B	109.5
N2—C9—C10	118.93 (13)	H23A—C23—H23B	109.5
C15—C10—C11	118.51 (14)	C20—C23—H23C	109.5
C15—C10—C9	122.35 (13)	H23A—C23—H23C	109.5
C11—C10—C9	119.15 (13)	H23B—C23—H23C	109.5
			
N1—Ni1—O1—C9	−1.22 (10)	O1—C9—C10—C11	1.6 (2)
N1^i^—Ni1—O1—C9	178.78 (10)	N2—C9—C10—C11	−179.24 (15)
O1^i^—Ni1—N1—C8	5.45 (14)	C15—C10—C11—C12	−0.3 (2)
O1—Ni1—N1—C8	−174.55 (14)	C9—C10—C11—C12	179.90 (14)
O1^i^—Ni1—N1—N2	−178.16 (10)	C10—C11—C12—C13	−1.1 (3)
O1—Ni1—N1—N2	1.84 (10)	C16—O2—C13—C12	172.90 (15)
C8—N1—N2—C9	174.67 (14)	C16—O2—C13—C14	−6.6 (2)
Ni1—N1—N2—C9	−1.98 (15)	C11—C12—C13—O2	−178.27 (15)
C6—C1—C2—C3	−1.5 (3)	C11—C12—C13—C14	1.3 (3)
C7—C1—C2—C3	178.71 (17)	O2—C13—C14—C15	179.45 (15)
C1—C2—C3—C4	2.2 (3)	C12—C13—C14—C15	0.0 (3)
C2—C3—C4—C5	−0.9 (3)	C13—C14—C15—C10	−1.4 (3)
C2—C3—C4—C8	178.31 (16)	C11—C10—C15—C14	1.5 (2)
C3—C4—C5—C6	−1.0 (3)	C9—C10—C15—C14	−178.65 (15)
C8—C4—C5—C6	179.76 (15)	C13—O2—C16—C17	−177.34 (14)
C4—C5—C6—C1	1.6 (3)	O2—C16—C17—C18	103.66 (19)
C2—C1—C6—C5	−0.4 (3)	O2—C16—C17—C22	−77.6 (2)
C7—C1—C6—C5	179.40 (16)	C22—C17—C18—C19	−0.6 (3)
N2—N1—C8—C4	0.3 (3)	C16—C17—C18—C19	178.15 (17)
Ni1—N1—C8—C4	176.52 (13)	C17—C18—C19—C20	0.6 (3)
C5—C4—C8—N1	−164.03 (17)	C18—C19—C20—C21	0.1 (3)
C3—C4—C8—N1	16.8 (3)	C18—C19—C20—C23	−178.8 (2)
Ni1—O1—C9—N2	0.40 (19)	C19—C20—C21—C22	−0.9 (4)
Ni1—O1—C9—C10	179.52 (10)	C23—C20—C21—C22	178.1 (2)
N1—N2—C9—O1	1.0 (2)	C18—C17—C22—C21	−0.2 (3)
N1—N2—C9—C10	−178.07 (12)	C16—C17—C22—C21	−178.91 (19)
O1—C9—C10—C15	−178.23 (14)	C20—C21—C22—C17	0.9 (4)
N2—C9—C10—C15	0.9 (2)		

Symmetry code: (i) −*x*+2, −*y*, −*z*+2.

### Hydrogen-bond geometry (Å, º)

**Table:**

*D*—H···*A*	*D*—H	H···*A*	*D*···*A*	*D*—H···*A*
C8—H8···O1^i^	0.95	2.38	2.9455 (18)	118
C3—H3···N2	0.95	2.37	2.945 (2)	118
C11—H11···O1	0.95	2.43	2.7590 (19)	100

Symmetry code: (i) −*x*+2, −*y*, −*z*+2.
